# Relationship between perceived coercion and perceived justification of coercive measures – secondary analysis of a randomized-controlled trial

**DOI:** 10.1186/s12888-023-05192-y

**Published:** 2023-10-02

**Authors:** Alexandre Wullschleger, Angelika Vandamme, Juliane Mielau, Andreas Heinz, Felix Bermpohl, Lieselotte Mahler, Christiane Montag

**Affiliations:** 1 Department of Psychiatry and Psychotherapy, Berlin Institute of Health, Charité Universitätsmedizin Berlin, Corporate Member of Freie Universität Berlin, Humboldt- Universität Zu Berlin, Campus Charité Mitte, Berlin, Germany; 2 Division of Adult Psychiatry, Department of Psychiatry, Geneva University Hospitals, Ch. Du Petit-Bel-Air 2, Thônex, 1226 Switzerland; 3 Department of Psychiatry, Clinics in the Theodor-Wenzel-Werk, Berlin, Germany

**Keywords:** Coercion, Inpatients, Mental health services

## Abstract

**Background:**

Subjective perception of coercion has gained attention as an important outcome. However, little is known about its relation to patients’ appraisal of the justification of coercive measures. The present study aims to analyze the relationship between patients’ appraisal of the justification of coercive measures and their level of perceived coercion.

**Methods:**

This study presents a secondary analysis of the results of a multi-center RCT conducted to evaluate the effects of post-coercion review. Patients who experienced at least one coercive measure during their hospital stay were included in the trial. Participants’ appraisal of the justification of coercive measures was categorized into patient-related and staff-related justifications. Subjective coercion was assessed using the Coercion Experience Scale (CES) and used as dependent variable in a multivariate regression model.

**Results:**

97 participants who completed the CES were included in the analysis. CES scores were significantly associated with the perception of the coercive measure as justified by staff-related factors (B = 0,540, p < 0,001), as well as with higher level of negative symptoms (B = 0,265, p = 0,011), and with mechanical restraint compared to seclusion (B=-0,343, p = 0,017).

**Conclusions:**

Patients’ perceptions of coercive measures as justified by staff-related factors such as arbitrariness or incompetence of staff are related to higher levels of perceived coercion. Multiprofessional efforts must be made to restrict the use of coercive measures and to ensure a transparent and sustainable decision-making process, particularly with patients showing high levels of negative symptoms. Such key elements should be part of all coercion reduction programs.

## Introduction

Although coercive measures are ethically justified in life-threatening clinical situations (such as severe delirium) or in most serious endangerment of others, their use is restricted to those situations where no other alternatives can be employed. Their potentially severe consequences, including a deterioration of the therapeutic relationship and the treatment course, subjective feelings of punishment and distress, the development of post-traumatic symptoms or physical injuries must be acknowledged and considered in decision-making processes [1, 2]. The raised ethical and human rights-related issues must be thoughtfully addressed in the development and evolution of policies and practices [3]. Interventions aiming at reducing the usage of coercion to an absolute minimum are the object of thorough research and developments in clinical practice [4, 5].

Besides the objective use of coercive measures, the aspect of subjective or perceived coercion has been highlighted as an important outcome in the context of psychiatric care. Subjective coercion can be defined as the patients’ perceptions, views and feelings related to their experience of coercion. High levels of perceived coercion are related to low patient satisfaction and negative attitudes towards hospital treatment [6], which are in turn associated with higher rates of readmission after one year [7]. Perceived coercion also negatively impacts the quality of the therapeutic relationship [8]. Among the different coercive measures, mechanical restraint has been shown to have a higher coercive potential than seclusion, and to be less accepted by patients [9, 10]. As to the specific burden associated with coercive measures, Steinert et al. showed that mechanical restraint was associated with a higher perceived burden after one year compared to seclusion [11].

Coercive measures are often perceived by patients as extremely humiliating and dehumanizing [12]. Results from qualitative studies strongly emphasize the importance of communication, respect of patients’ rights and transparency in decision-making to reduce the use of coercion and to alleviate the strong negative feelings associated with such measures in psychiatric care [13, 14]. The quality of the relationship and a respectful communication with caregivers as well as aspects of the physical environment of wards such as intimacy and comfort seem to linger the subjective experience of coercion as well [15]. Authors also emphasized the importance of staff’s appraisal of the patients’ perception of coercion [16]. Perception of fairness during the admission and treatment process and feelings of being treated with respect seem to reduce the level of subjective perception of coercion [17–19]. The perception of coercion as a violent rupture of the therapeutic relationship that could only be explained by an arbitrary or even unethical abuse of power can be devastating and lead to dramatic subjective and interpersonal consequences, like intense feelings of despair, distress and even the development of post-traumatic symptoms [1, 12]. However, little is known about the way patients’ appraisal of decision-making processes regarding coercive measures and how this might influence their level of perceived coercion.

The present work aims at investigating the potential relationship between patients’ appraisal of the justification of coercive measures and the level of subjective distress they experienced during their application. It was hypothesized that the perception of coercive measures as justified by arbitrariness or other factors solely related to staff members or structural issues would increase the experienced distress.

## Methods

We performed a secondary analysis of a multi-center, two-armed, randomized controlled trial assessing the effect of standardized post-coercion review on posttraumatic symptoms and subjective coercion (ClinicalTrials.gov ID NCT03512925). The specific design of this RCT has been described in detail in previous publications [20, 21]. The RCT was conducted in six clinics in the region of Berlin, all providing acute psychiatric care for a defined catchment area between November 2017 and May 2019.

### Coercive measures

Coercive measures were defined as mechanical restraint, seclusion or forced medication. In Germany, these measures can be applied by psychiatrists in case of acute endangerment of self or others. Seclusion or restraint measures lasting longer than 18 h must be authorized by the Civil Court. As to forced medication outside of an emergency, it must also be authorized by the Court and must be limited in time.

### Participants

Participants were recruited in six psychiatric hospitals providing acute psychiatric care for a defined catchment area, on inpatient wards where coercive measures were performed. Inclusion criteria were: age between 18 and 65, diagnosis of a psychotic disorder (ICD-10: F1x.5, F2x, F30.2, F31.2), and documented experience of at least one coercive measure such as mechanical restraint, seclusion or coerced medication on court order during their hospital stay. We excluded patients who were discharged within 24 h, who presented severe cognitive deficits, or who only had limited knowledge in German.

As specified in the previous articles, potential participants, who were not able to give their consent when the first coercive measure took place, were included in the trial following a Zelen’s design, which allows to randomize participants without their explicit consent when the foreseen intervention only minimally differs from routine care, as was the case here [22, 23]. Participants were then contacted before discharge by members of the research team not involved in their care and informed about the study procedures. They were then offered the possibility to consent to the assessment, which was conducted by the research team’s members.

### Measures

#### Socio-demographic and clinical data

The following variables were obtained during assessment by the interviewers: age, gender, level of education, history of migration, if the person receives incapacity benefits, and past experiences of coercive measures. As to the clinical variables, level of functioning was assessed with the GAF (Global Assessment of Functioning) and global severity of symptoms with CGI-S (Clinical Global Impression - Severity scale). The level of positive and negative symptoms as well as the level of insight were assessed using four-point Likert scales (absent, mild, moderate, severe) to simplify the evaluation of symptoms and limit missing data. All clinical variables were obtained from the psychiatrists in charge of the patients at the time of discharge.

**Table 1:** Socio-demographic and clinical characteristics of the studied sample

Age (yrs) *M (SD)*	38.49 (12.88)
**Gender**	
Female n (%)	49 (50.5%)
Male n (%)	48 (49.5%)
**Hist. of migration** n (%)	*n = 91*
Yes n (%)	22 (24.2%)
No n (%)	69 (75.8%)
**Incap. benefits** n (%)	*n = 87*
Yes	25 (28.7%)
No	62 (71.3%)
**Level of education** n (%)	*n = 86*
No degree	4 (4.7%)
Lower sec. education	13 (15.1%)
Higher sec. education	24 (27.9%)
High school graduation	15 (17.4%)
Vocational college	13 (15.1%)
University	17 (19.8%)
**Diagnosis** n (%)	
F19.x5, F30.2, F31.2	22 (22.7%)
F2.x	75 (77.3%)
**Clinical parameters**	*n = 88*
GAF *M (±SD)*	27.44 (13.37)
CGI-S *M (±SD)*	5.67 (0.69)
*Symptom severity M (±SD)*	
Positive sympt.	2.35 (0.89)
Negative sympt.	1.19 (0.91)
Lack of insight	2.34 (0.88)
**Past coercion** n (%)	*n = 96*
yes	65 (67.7%)
no	31 (32.3%)
**Index coercive measure n (%)**	
Restraint	61 (62.9%)
Seclusion	29 (29.9%)
Forced med. on court order	7 (7.2%)
**Number of coercive events** ***M (±SD)***	
Restraint	1.90 (2.24)
Seclusion	2.07 (2.33)
**Time between coercive measure and assessment (days)** ***M (±SD)***	45.33 (30.96)
**Duration of stay (days) median** ***(±IQR)***	53 (62.5)
**Coercion Experience Scale** *M (±SD)*	2.99 (0.97)
**Remembrance of the coercive measure** *M (±SD)*	67.89 (32.62)

CGI-S: Clinical Global Impression – Severity Scale; CGI-I: Clinical Global Impression – Improvement Scale; GAF: Global Assessment of Functioning; M: mean; SD: standard deviation

#### Subjective coercion experienced during coercive measures

The Coercion experience Scale (CES) [24] was used to assess the subjective impact of the first experienced coercive measure that initiated the randomization process. The CES is a 32-item, self-rating instrument featuring patients’ perspective on harmful aspects of coercive measures. A standardized ratio of the total score was used, as suggested by the authors. An additional item, rating the capacity of the person to remember the coercive measure on a 0-100 analog scale (self-rating), was also featured in the evaluation and used in the analysis as covariate.

#### Justification of coercive measures

Participants of the trial were asked to rate the reasons they retrospectively thought to have motivated the use of the first coercive measure. Participants were presented with 11 possible justifications for the use of coercion. These were derived from a previous study investigating patients’ views of the reasons leading to coercive interventions [19]. They were asked to rate their endorsement of each item on a four-point Likert scale ranging from 1 (no) to 4 (yes). Reasons were then categorized for the analysis in two main categories: reasons related to the participant’s clinical state (six items: acute distress, acute necessity of treatment, endangerment of oneself, disinhibited behavior, restlessness, endangerment of others) and reasons related to the staff (five items: incompetence of staff, arbitrariness, punishment, lack of alternatives on the ward, lack of staff). Staff members include all professionals working on wards (psychiatrists, nurses, psychologists, etc.). Standardized ratios of the constituting items were used to build the final scores of the two main categories, patient-related and staff-related justifications, with possible values ranging from 1 to 4. Higher values indicate a stronger endorsement of the justifications.

Both CES and rating of the justification for the experienced coercive measure were assessed together at discharge.

### Statistical analysis

Potential influencing factors of the level of perceived coercion as measured by the CES were tested in bivariate linear regression analysis. Factors correlating with CES scores at a p < 0,1 level were included as predictors in a multiple linear regression model using CES scores as dependent variable.

Because of the small number of participants (n = 8; 7,3% of the total sample) who were submitted to forced medication, we chose to exclude these from analysis.

Statistical analysis was performed using IBM SPPS Statistics 25. Significance was defined at a two-sided *p* < 0.05.

## Results

The description of the sample’s socio-demographic and clinical variables is summarized in Tables 1 and 2. N = 97 patients who completed the CES were included in the analysis. The mean CES score was 2,99 (SD ± 0,97).

**Table 2:** Participants’ appraisal of the justification for the use of coercion

Reasons	Mean (±SD)
Patient-related	
Acute distress	2.29 (1.31)
Acute necessity of treatment	2.45 (1.34)
Endangerment of oneself	1.58 (1.09)
Disinhibited behavior	2.19 (1.29)
Restlessness	2.63 (1.35)
Endangerment of others	2.00 (1.27)
**Staff-related**	
Incompetence of staff	2.44 (1.31)
Arbitrariness	2.07 (1.25)
Punishment	1.77 (1.19)
Lack of alternatives	2.32 (1.34)
Lack of staff	1.94 (1.27)

Results of the bivariate regression analyses are displayed in Table 3. The following variables were included in the multivariate analysis: type of coercive measure, perception of staff-related reasons for the coercive measure, negative symptoms, lack of insight, CGI-S, and age.

**Table 3:** Results of the bivariate regression analyses

	Bivariate regression				
	B	Beta	95% CI		*p*
Female gender	0.72	0.037	-0.339	0.482	0.729
Age	-0.014	-0.177	-0.030	0.002	0.095*
Mechanical restraint	-0,511	-0.247	-0.937	-0.086	0.019*
GAF	-0.006	-0.087	-0.022	0.009	0.437
CGI-S	0.328	0.241	0.034	0.622	0.029*
CGI-I	-0.057	-0.041	-0.370	0.256	0.717
Positive symptoms	0.068	0.062	-0.174	0.310	0.578
Negative symptoms	0.272	0.249	0.037	0.508	0.024*
Lack of insight	0.215	0.200	-0.011	0.440	0.062*
Patient-related justification	-0.033	-0.028	-0.282	0.217	0.795
Staff-related justification	0.600	0.519	0.389	0.810	< 0.001*
Past coercion	-0.091	-0.043	-0.537	0.355	0.686
Study center	0.099	0.165	-0.026	0.224	0.120
Remembrance of the measure	-6.653	-0.002	-0.006	0.006	0.983
Psychotic disorder	0.022	0.010	-0.455	0.499	0.927
Time between coercive measure and assessment	0.003	0.105	-0.003	0.10	0.305

CI: Confidence Interval; CGI-S: Clinical Global Impression – Severity Scale; GAF: Global Assessment of Functioning. *p < 0.1

A significant regression equation was found (*F* (6,81) = 8.273, *p* < 0.001), explaining 38% of the variation of CES scores (R^2^ = 0,380). Results of the multiple linear regression model are displayed in Table 4.

**Table 4:** Results of the multiple linear regression model

	Multivariate model					
	B	Beta	95% CI		*t-test*	*p*
Age	0.004	0.051	-0.011	0.019	0.524	0.601
Coercive measure						
Seclusion (ref. category)	-	-	-	-	-	-
Mechanical restraint	-0,343	-0.226	-0.625	-0.062	-2.428	0.017*
CGI-S	0.108	0.079	-0.155	0.371	0.818	0.416
Negative symptoms	0.265	0.254	0.063	0.468	2.612	0.011*
Lack of insight	0.065	0.060	-0.011	0.019	0.524	0.601
Staff-related justification	0.540	0.498	0.343	0.738	5.454	< 0.001*

CI: Confidence Interval; .CGI-S: Clinical Global Impression – Severity Scale. *p < 0.05

Perception of the coercive measure as justified by staff-related factors (B = 0,540, p < 0,001) was significantly associated with CES scores, which confirmed our initial hypothesis. The type of coercive measures (B=-0,343, p = 0,017) and the severity of negative symptoms (B = 0,265, p = 0,011) were also significant predictors of the CES scores. The perception of the coercive measure as motivated by staff-related reasons was associated with higher CES scores. Mechanical restraint was also associated with higher CES scores, as was a higher severity of negative symptoms. Severity of symptoms as measured by the CGI-S and age were not significantly associated with CES scores. Level of insight was not correlated as well with CES scores.

## Discussion

The results of our analysis show that the psychological impact of coercive measures like seclusion and restraint is significantly influenced by the perception of the reasons that led to their use. This finding offers new insights in the determinants of patients’ perception of coercion.

In our model, the subjective burden caused by seclusion and restraint was significantly increased when patients perceived that the coercive measure was decided on the basis of factors related to staff members and hospital structures. The experience of coercion as arbitrary, as punishment, as a result of staff incompetency or as resulting from a lack of alternatives seems to aggravate the distress associated with these measures. This in line with previous studies underlining the importance of perceived fairness and adequacy of hospital treatment for the overall perception of coercion [15]. This experience of arbitrariness is particularly central and should be emphasized as a key element in the experience of coercion. Our finding thus reminds us of the uttermost importance of a thorough and thoughtful interprofessional decision-making process when considering the use of seclusion or restraint measures. This ensures that coercion is only applied as a last resort after all alternatives have been considered. It also helps involved staff members being collectively responsible for the decision made and able to communicate this decision as the result of this process to the patient. A high degree of transparency, including the disclosure of the decision-making processes of staff members before and during all stages of the coercive measure might enable the patient to remain in dialogical contact and not view the measure as merely arbitrary or punitive, as was shown by previous works [25]. Shared-decision making interventions have shown interesting results regarding involuntary hospital admissions [26]. New specific interventions in the context of coercive measures might thus be of interest. Surely, this will not prevent patients from experiencing distress during seclusion of restraint events but should be an important requisite to reduce this burden. Advance statements such as advance directives or crisis plans can also surely be helpful in this context as tools aiming to reduce the use of coercive measures, as literature has shown the promising potential of such instruments to reduce coercion [27, 28].

As to the other significant determinants of the perceived coercion as measured by the CES, our results confirm previous studies showing that restraint causes greater distress than seclusion [11]. When also considering the literature showing the deleterious physical consequences of mechanical restraint, the present study further underlines the need to strictly control the use of such measures and to associate them with close surveillance and therapeutic attention, when other less coercive and invasive measures are not available. Interestingly, when patients were asked whether they thought restraint was warranted in fictional scenarios of psychiatric practice, they were just as likely as healthy individuals to recommend restraint and the forced administration of medication, but significantly less likely to endorse restraint as an appropriate measure, and the acceptance of coercive measures in general was reduced, when patients had experienced lack of effectiveness and transparency in their own treatment [19].

Interestingly, the level of negative symptoms significantly influenced the perception of the coerciveness of seclusion and restraint. It is possible that patients showing greater negative symptoms, probably linked to more prominent, disease-related cognitive impairments, are more vulnerable to the psychological impact of coercive measures and have difficulties to understand and interpret the context of the interventions. This is line with a recent research that showed that negative symptoms were associated with a lower feeling of dignity in inpatient care [29]. Another work by Horvath et al. (2018), also showed that lower levels of insight and higher levels of negative symptoms were both correlated with a higher perceived coercion regarding treatment in forensic setting [30]. Moreover, negative symptoms like withdrawal, social anhedonia or reduced affective expressivity may also serve a protective function for people with interpersonal anxieties or fragile ego boundaries, which then would be overrun by coercion. It is thus of great importance to show a special attention to patients with high levels of negative symptoms, as their higher perception of coercion might negatively influence adherence to treatment, satisfaction with care and the further course of treatment [6, 8].

Our results are drawn from the secondary analysis of data gathered during an RCT evaluating the effects of standardized post-coercion review on symptoms of PTSD and subjective coercion. Previous results of this RCT showed that the intervention had a positive effect on PTSD symptoms [20]. As to subjective coercion, the analysis showed that the intervention had a positive effect on the perception of coercion in women [21]. Our analysis yielded no specific effect of the intervention on CES scores, most probably because of the inability of the intervention to retrospectively influence the distress caused by coercive measures. However, the present results and the shown effect of post-coercion review on subjectively perceived coercion indicate that such an intervention might be useful to address the question of the justification of coercive measures and to help repair the damaged therapeutic relationship through its joint analysis of events and its focus on transparency and accountability of the decision-making process.

Some limitations of the present work must be considered. First, there is to some extent a semantic overlap between items of the CES and the items used to assess the justification of coercive measures as perceived by patients, as some items of the CES are somehow related to the justification of the coercive measure. This overlap may have had an influence on the results. This limitation is directly linked to the relative lack of more precise tools and scales assessing subjective coercion that might better reflect certain aspects of patients’ experiences. Future research should address this issue. Participative research designs would certainly be helpful in this matter. Second, the assessment of distress associated with experienced coercion only concerns the first coercive measure experienced by patients and thus does not consider multiple occurrences of coercive measures. This was linked to the post-coercion review intervention (focusing only on one coercion event) that was the object of the RCT. Third, our assessment did not include previous traumas that might increase the distress experienced during coercive measures. Fourth, the assessment of symptoms was made using a simple Likert scale and wasn’t based on validated scales like the PANSS, which might have underestimated the burden of psychotic symptoms. Lastly, the questions used to assess the justification of coercive measures were not based on a validated questionnaire, although derived from a previous investigation [19]. Staff members’ concomitant appraisal of the justification of coercive measures was also not assessed in the present work and could be an interesting investigation field in further studies. The presented results should thus be interpreted with caution considering these limitations.

Despite these limitations, the present work is the first to have specifically studied the influence of the perception of the justification of coercive measures on the distress they cause. The relatively large sample allowed for a robust analysis and results give new insights into the experience of coercion among severely ill patients in hospital setting.

In conclusion, the present study indicates that specific attention should be given to the decision-making and communication process, including the use of shared decision-making tools surrounding coercive measures. Existing clinical models that emphasize transparency and participation and, in particular, make decision-making processes transparent should be further implemented [31]. A thorough team analysis of potential alternatives, a respectful and transparent application of coercion and the absolute respect of patients’ basic rights are mandatory to help alleviate the very high level of distress experienced by patients. It may also be of great importance to specifically address these issues during mechanical restraint and by patients with high levels of negative symptoms who might be confronted to more negative and distressful experiences.

## Data Availability

Orignial data of the present study is available from the corresponding author upon reasonable, motivated request.
